# The association between physical activity and sleep in adult ADHD patients with stimulant medication use

**DOI:** 10.3389/fpsyt.2023.1236636

**Published:** 2023-11-20

**Authors:** Feilong Zhu, Boya Liu, Dongqing Kuang, Xiaotong Zhu, Xiaoyu Bi, Yiqi Song, Tianshen Quan, Yiming Yang, Yuanchun Ren

**Affiliations:** ^1^College of Physical Education and Sports, Beijing Normal University, Beijing, China; ^2^University of Southern California, Los Angeles, CA, United States

**Keywords:** attention-deficit/hyperactivity disorder (ADHD), stimulants, sleep, physical activity, NHANES

## Abstract

**Background:**

Adults with attention-deficit/hyperactivity disorder (ADHD) may experience sleep problems doubly suffering from the disease and side effects of stimulant medications. Physical activity (PA) is known to produce numerous beneficial effects in adults. However, it was not well-characterized whether PA would still be effective in this situation. The main objective of the current study was to examine the relationship between PA and sleep among adult ADHD patients who were using stimulant medications and quantify the form of this association.

**Methods:**

Adult ADHD participants with stimulant medications use condition from the National Health and Nutrition Examination Survey (NHANES) database between January 1, 2013, and March 2020 (prepandemic) were included in the cross-sectional analysis. Weighted logistic regression was performed to assess the relationship between PA level and sleep. A restricted cubic spline model was used to relax the linear relationship assumptions and investigate the associations between the risk of trouble sleeping and time spent engaging in moderate-to-vigorous PA per week.

**Results:**

A total of 162 eligible adult ADHD participants who reported using stimulant medicines were included. Participants who adhered to the general recommendation of guidelines in the US of 150 min per week of moderate-to-vigorous PA had a significant lower risk of complaining of trouble sleeping (OR: 0.26, 95% CI: 0.10–0.67, *p* = 0.006), and this association was seen in men (OR: 0.23, 95% CI: 0.09–0.56, *p* = 0.002), but was not seen in women (OR: 0.71, 95% CI: 0.27–1.88, *p* = 0.500). Restricted cubic spline analysis showed that the incidence of trouble sleeping gradually decreased after at least 105 min of moderate-intensity PA per week in participants (OR: 1.02, 95% CI: 0.92–1.14). A significant difference appeared after 341 min (OR: 0.87, 95% CI: 0.76–0.99), and the curve leveled after 1,250 min (OR: 0.60, 95% CI: 0.46–0.79).

**Conclusion:**

Our findings observed associations between PA and sleep condition in the adult ADHD patients with stimulant medication use population. Moderate-to-vigorous PA may be beneficial to sleep in adults with ADHD who were using stimulants and thus should be recommended as part of a healthy lifestyle. Gender difference should be considered as an important factor for further studies to examine these associations and explore potential mechanisms.

## Introduction

Attention-deficit/hyperactivity disorder (ADHD) is a common neurodevelopmental psychiatric disorder in childhood that is characterized by inattention, hyperactivity, and/or impulsivity, which cause functional impairment and lead to difficulties in academic achievement and daily life (1). The prevalence of ADHD in children and adolescents is estimated to be 5.29 to 7.2% around the world, and this number is above 10% in China (2). Furthermore, ADHD is not confined to childhood or adolescence, with nearly 60% of children with ADHD experiencing symptoms into adulthood, causing disruptions at work and in personal relationships (3). The prevalence of ADHD in adults has been shown to be 2.5% in a systematic review of epidemiological studies (4) and 2.8% in the most current World Health Organization (WHO) Mental Health Survey across 10 countries (5). It is worth noting that the incidence of adult ADHD is likely to be underestimated because ADHD is often considered a childhood disorder that improves with age, and the fourth edition of the Diagnostic and Statistical Manual of Mental Disorders (DSM-IV) criteria is insensitive to the adult population (4, 5). Therefore, adult ADHD should attract much attention.

Executive function impairments are a defining trait among individuals with ADHD and are a current focus of academic research. Additionally, ADHD has been associated with sleep problems, similar to other neurodevelopmental disorders. It is estimated that up to 85% of adults with ADHD have sleep disturbances (6). These disturbances can manifest as long sleep latency, difficulty falling and staying asleep, altered total sleep duration, sleep phase delay syndrome, increased periodic limb movements when sleeping, and drowsiness during the day (7, 8). Fuller-Thomson et al. reported a substantial link between ADHD and insomnia, with those who self-reported having ADHD being five times more likely to suffer from insomnia (OR = 5.18) than those without ADHD (9). Previous studies have reported that sleep deprivation exerts multiple effects on neurobehavioral and cognitive systems, including attention and emotional regulation. As a result, sleep disturbances in ADHD may affect the core psychopathology of the condition. In recent times, there has been an increasing number of adults diagnosed with ADHD and receiving prescribed stimulant medications treatment (10, 11). Methylphenidate, a central nervous system stimulant, has been tested extensively in clinical trials on adults with ADHD, but the overall benefits and risks remain unclear due to concerns about how these trials were designed and how the results were reported. Many previous research results revealed that stimulant medications have been shown to provide marked benefits in symptoms of ADHD, but cause disruptions in circadian rhythms and sleep that may negatively affect mood regulation (12–14). Some researchers have also noted that contrary to sedatives and hypnotics, which reduce brain activity and increase sleepiness, stimulants affect the body and central nervous system, inducing heightened alertness, difficulties falling asleep and delay of circadian rhythmicity (13, 14). In general, adult ADHD patients who take stimulant medicines are particularly vulnerable because they are frequently ignored and also may experience sleep problems doubly suffering from the disease and stimulant medications.

Physical activity (PA) has been shown to improve sleep quality for many people. Specifically, moderate-to-vigorous exercise can improve the quality of sleep for adults by speeding up the process of falling asleep and decreasing the amount of time spent awake in bed at night. Moreover, engaging in PA can alleviate daytime sleepiness and reduce the need for sleep medications for some people. The mechanisms by which PA improves sleep include mild body warming after exercise, improvement of vagus nerve function (the vagus nerve mainly plays an inhibitory role in promoting sleep), regulation of cortisol and other endocrine hormone fluctuations, and mood enhancement resulting in relaxation (15). The PA guidelines from the US and WHO recommend that adults engage in moderate-intensity PA for at least 150 min/week or vigorous PA for ≥75 min/week (16). Despite the common perception that people with ADHD are sufficiently active or even hyperactive, they may not engage in optimal movement behaviors for health (17).

Collectively, preliminary findings supported that adults with ADHD may experience sleep disturbs suffering from both the disease and stimulant medications. However, it was not well-characterized whether PA would still be effective in this situation. It remained doubtful that what is the relationship between PA and sleep in the adult ADHD patients with stimulant medication use group. To our knowledge, no public studies have examined the impact of PA on sleep in adult ADHD stimulant users. Therefore, we believe this is an important clinical question worthy of further study for this ‘special group’. In this study, by using a general sample from the National Health and Nutrition Examination Survey (NHANES) in the US, we sought to examine the association between PA and sleep in the adult ADHD patients with stimulant medication use group and quantify the form of this association.

## Methods

### Study design and sample

The current study’s data came from the National Health and Nutrition Examination Survey (NHANES), which was a nationally representative complex, stratified, and multistage probability sample of adults and children in the United States. The survey is distinctive in that it combines interviews, physical examinations, and laboratory testing conducted in participants’ homes and in a traveling mobile examination center (MEC). NHANES conducts laboratory tests such as blood tests, urine tests, nutritional assessments, and evaluations for environmental exposures. NHANES data are freely accessible on the website^1^ for researchers worldwide. The National Center for Health Statistics of the Centers for Disease Control and Prevention Institutional Review Board approved the protocol for the original NHANES data collection, and informed consent was acquired from all individual participants.^2^

The sample with adults over 20 years old included in three cycles (2013–2014, 2015–2016, and 2017-March 2020 prepandemic) of NHANES were chosen for data analysis because they included information about demographics, anthropometrics, prescription medicine use, sleep, physical activity and other covariates used in this study.

### Measurements

#### Stimulant medicine use and diagnosis of ADHD

Data on medication use were collected through a medication questionnaire conducted by trained interviewers using the Computer-Assisted Personal Interview (CAPI) system. Participants were asked whether they had taken any prescription medications during the month preceding the interview date. Specific questions included “Have you used or taken medication that requires a prescription in the last 30 days? Do not add any prescription vitamins or minerals that you may have already mentioned to me.” In addition, participants were asked to provide the generic name of the drug, how long they had been taking it, their main reason for taking it, and any ICD-10-CM (International Classification of Diseases, Tenth Revision, Clinical Modification) codes related to their reported reasons for use. Data were periodically examined for discrepancies and incorrect entries for quality assurance and control. The interviewer’s list of drugs was compared to the medication names selected from the drug database. Stimulants in this study were defined as methylphenidate, amphetamine, dexmethylphenidate, dextroamphetamine, lisdexamfetamine dimesylate, and levoamphetamine. Based on stimulant medication use and ICD-10-CM codes, participants with ADHD using stimulant medications were identified.

#### Assessment of sleep parameters

The sleep parameters were derived from the “sleep disorders” dataset comprising questions on sleep habits and disorders in the “NHANES Questionnaire.” Sleep duration was recorded by answering the question “The number of hours that you typically sleep on weekdays or workdays,” and hours were rounded to the closest half-hour (18). Sleep duration was categorized as short (<7 h per night), normal (7–9 h per night) and long (>9 h per night) (19, 20). The answer to the question “Have you ever mentioned your difficulty sleeping to a doctor or other health care provider?” was used to assess trouble sleeping. As shown in Table 1, healthy sleep scores and patterns were established using sleep characteristics such as sleep duration, trouble sleeping, snoring, snorting or stopping breathing, and feeling excessively sleepy during the day. Sleep total scores of 0–1, 2–3, and 4–5 indicated poor, intermediate, or healthy sleep patterns, respectively (21).

**Table 1:** Definition of a healthy sleep score and sleep patterns.

Sleep factors	Score
*Sleep duration (hour)*	
<7	0
7–9	1
>9	0
*Trouble sleeping*	
No	1
Yes	0
*Snoring*	
Never	1
Rarely/occasionally/frequently	0
*Feeling overly sleepy during the day*	
Never/rarely	1
Sometimes/often/almost always	0
*Snorting or stopping breathing*	
Never	1
Rarely/occasionally/frequently	0
*Sleep patterns*	Total scores
Healthy sleep	4–5
Intermediate sleep	2–3
Poor sleep	0–1

#### Physical activity

The Global Physical Activity Questionnaire (GPAQ) was used to evaluate physical activity. The questionnaire consisted of 6 questions pertaining to leisure time physical activity, which were administered as part of the home interview. Participants were asked if they regularly participated in PA by the following questions: (1) “In a typical week, do you do any vigorous-intensity sports, fitness, or recreational activities that cause large increases in breathing or heart rate, such as running or basketball for at least 10 min continuously?” and (2) “Do you engage in any moderate-intensity sports, fitness, or recreational activities such as brisk walking, bicycling, swimming, or volleyball for at least 10 min continuously during a typical week?” Those who responded “yes” to either question were subsequently queried regarding the number of days and amount of time spent in each activity intensity during a typical week. If a participant completed 150 min per week of moderate-intensity physical activity (MPA), 75 min per week of vigorous-intensity physical activity (VPA), or a combination of MPA and VPA that amounted to 150 min per week (with 1 min of VPA weighted as double that of MPA), they were deemed to have met the physical activity guideline and defined as the active group. On the contrary, participants were defined as the inactive group. This guideline was based on US national physical activity recommendations (22, 23).

#### Covariates

The study’s demographic covariates of interest included age (20–29 years, 30–44 years, 45–59 years, and ≥ 60 years), sex (female and male), race (Mexican American, Other Hispanic, Non-Hispanic White, Non-Hispanic Black, and Other), education level (high school or below and above high school), marital status (married/living with partner, widowed/divorced/separated, and never married), and income. Income was measured using the poverty-income ratio (PIR), which is the ratio of household income to the appropriate poverty threshold for household size (24). PIR was categorized as <2 (poor or near poor) and ≥ 2 (middle to high income) (25). Body mass index (BMI) was collected by physical examinations in the Mobile Examination Center, calculated as weight in kilograms divided by height in meters squared (kg/m^2^), and classified into <25, 25–30, and > 30 kg/m^2^ categories. Smoking status was determined by the question “Have you ever smoked more than 100 cigarettes in your life?” and classified as ‘yes’ or ‘no’. Daily alcohol intake was assessed from the total nutritional intake (DR1TOT) consumed for the 24 h before the interview, which was then separated into two categories: 0 and > 0 gm. Diabetes and hypertension were identified based on self-reported and medical diagnoses. The Patient Health Questionnaire-9 (PHQ-9) was used to assess the frequency of depressive symptoms over the previous 2 weeks in the NHANES. It is a nine-item depression screening tool with acceptable reliability and validity (26). Response categories for each item were scored on a 0–3 scale (0 = “not at all,” 1 = “several days,” 2 = “more than half the days,” and 3 = “nearly every day”), and the total PHQ-9 score ranged from 0 to 27, with higher PHQ-9 scores indicating a higher probability of severe depression. A total score of ≥10, according to the DSM-IV, was considered clinically relevant depression (26).

### Statistical analysis

Participants were divided into two groups as active and inactive groups. Continuous variables, such as age and BMI, were translated into categorical variables. Baseline data were analyzed using the chi-square test or Fisher’s exact test and expressed as the number (percentage). The odds ratios (ORs) and 95% confidence intervals (95% CIs) were calculated using a weighted logistic regression model with adjustment for covariates among baseline characteristics and stratified by gender to examine the associations between physical activity level and sleep in adult ADHD participants with stimulant use. We used a restricted cubic spline model to relax the linear relationship assumptions and investigate the associations between risk of trouble sleeping and time of moderate to vigorous PA (with 1 min of VPA weighted as double that of MPA) per week. The reference category for this analysis was without moderate to vigorous PA every week. R software, version 4.2.1 (The R Foundation for Statistical Computing), was used to execute every statistical analysis. *p* values less than 0.05 (*p* < 0.05) indicated statistically significant differences.

## Results

### The baseline characteristics of the study population

A total of 162 eligible ADHD participants who reported stimulant use (53.1% males and 46.9% females, mean (SD) age 39.1 [14.4] years) were included in this analysis. The characteristics of the participants according to physical activity level were presented in Table 2. There were no significant differences in sex, race, household income, smoking, diabetes, depressive symptoms and duration of stimulant use between two groups in the analytic sample. However, age distribution of the active group differed from inactive group, with the active group being younger. Compared to inactive group, the active group were more likely to be highly educated, never married, low obesity rate, alcohol intake, and had a lower prevalence of hypertension. Furthermore, there were significant differences in prevalence of trouble sleeping (*p* = 0.004) between two groups and no significant differences were found in sleep duration and sleep patterns.

**Table 2:** Characteristics of the adult ADHD stimulant users stratified by physical activity level.

Characteristics	Inactive group (*n* = 101)	Active group (*n* = 61)	*p* value
*Age (year)*			<0.001
20–29	22 (21.8%)	28 (45.9%)	
30–44	37 (36.6%)	24 (39.3%)	
45–59	27 (26.7%)	7 (11.5%)	
≥60	15 (14.9%)	2 (3.3%)	
*Sex*			0.599
Male	52 (51.5%)	34 (55.7%)	
Female	49 (48.5%)	27 (44.3)	
*Race*			0.915
Mexican American	10 (9.9%)	4 (6.6%)	
Other Hispanic	7 (6.9%)	3 (4.9%)	
Non-Hispanic White	63 (62.4%)	40 (65.6%)	
Non-Hispanic Black	10 (9.9%)	6 (9.8%)	
Other	11 (10.9%)	8 (13.1%)	
*Education level*			<0.001
High school or below	39 (38.6%)	8 (13.1%)	
Above high school	62 (61.4%)	53 (86.9%)	
*Marital status*			<0.001
Married/Living with partner	55 (54.5%)	23 (37.7%)	
Widowed/Divorced/Separated	24 (23.8%)	6 (9.8%)	
Never married	22 (21.8%)	32 (52.5%)	
*PIR* ^a^			0.585
<2	46 (47.4%)	24 (42.9%)	
≥2	51 (52.6%)	32 (57.1%)	
*BMI (kg/m^2^)* ^a^			0.007
<25	25 (26.3%)	29 (50.9%)	
25–30	53 (55.8%)	19 (33.3%)	
>30	17 (17.9%)	9 (15.8%)	
*Smoking status*			0.622
No	44 (43.6%)	29 (47.5%)	
Yes	57 (56.4%)	32 (52.5%)	
*Alcohol intake (gm/day)* ^a^			<0.001
0	66 (81.5%)	28 (53.8%)	
>0	15 (18.5%)	24 (46.2%)	
*Hypertension*			0.042
No	66 (65.3%)	49 (80.3%)	
Yes	35 (34.7%)	12 (19.7%)	
*Diabetes* ^a^			0.288
No	89 (88.1%)	59 (96.7%)	
Yes	10 (9.9%)	2 (3.3%)	
*Depressive symptoms* ^a^			0.701
<10	53 (66.2%)	27 (62.8%)	
≥10	27 (33.8%)	16 (37.2%)	
*Duration of stimulant use (year)*			
<1	8 (7.9%)	8 (13.1%)	0.283
≥1	93 (92.1%)	53 (86.9%)	
*Sleep duration (hour)*			0.872
<7	38 (37.6%)	21 (34.4%)	
7–9	52 (51.5%)	32 (52.5%)	
>9	11 (10.9%)	8 (13.1%)	
*Trouble sleeping*			0.004
No	30 (29.7%)	32 (52.5%)	
Yes	71 (70.3%)	29 (47.5%)	
*Sleep patterns* ^a^			0.138
Healthy sleep	9 (12.5%)	5 (12.5%)	
Intermediate sleep	38 (52.8%)	28 (70.0%)	
Poor sleep	25 (34.7%)	7 (17.5%)	

a Totaled numbers do not equal those in the column heads due to missing data.

### Association between physical activity and sleep in adult ADHD patients with stimulant use

**Figure 1:**
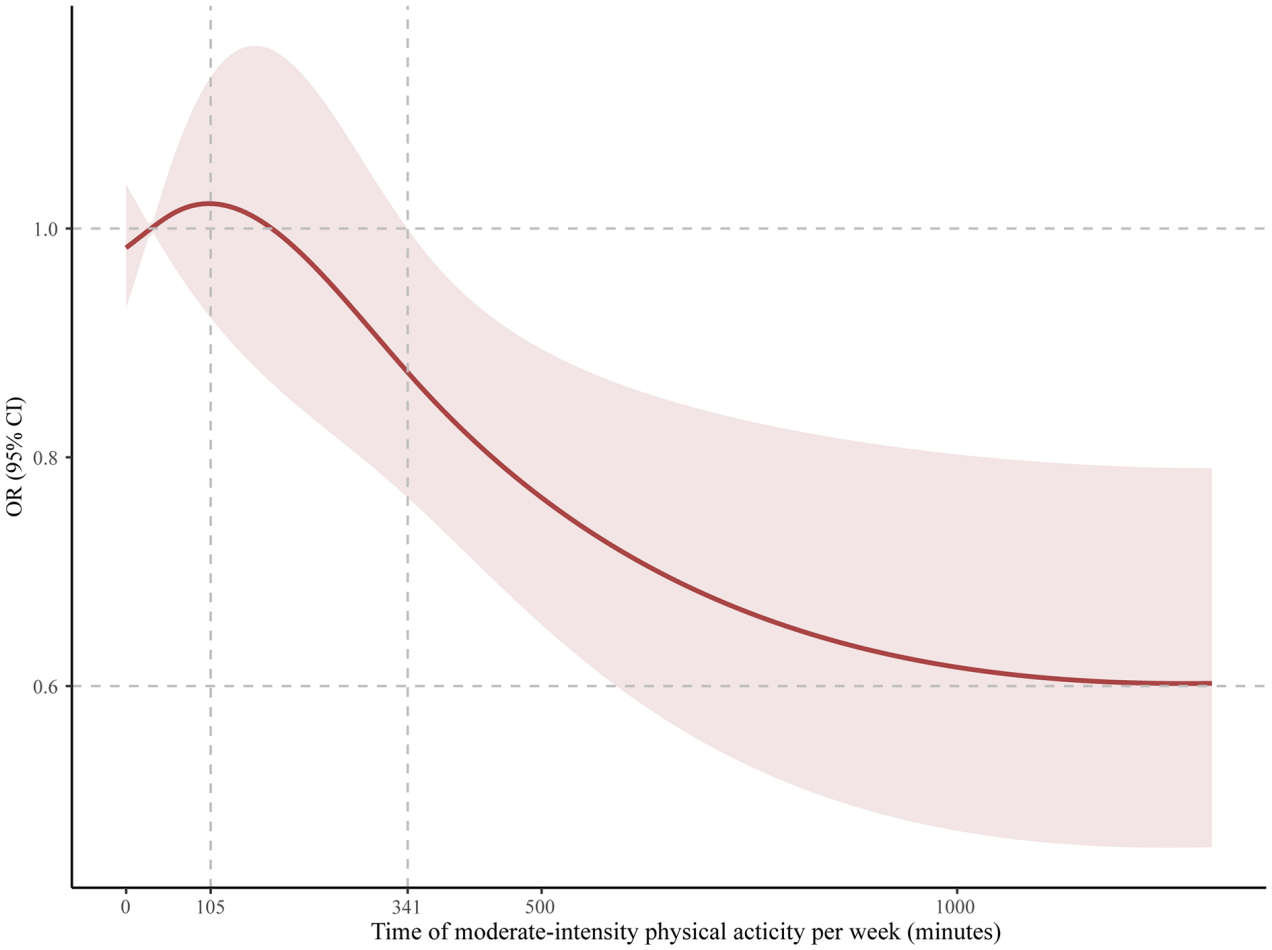
Odds ratio and moderate-intensity physical activity time per week for trouble sleeping in adult ADHD patients on stimulants. The reference group was 0 min/week.

Of the adult ADHD patients with stimulant use, 61 (37.7%) participants met the general recommendation of physical activity guidelines in the US. Patients who met the recommended 150 min/week of moderate to vigorous physical activity had a significantly lower risk of complaining of trouble sleeping (OR: 0.26, 95% CI: 0.10–0.67, *p* = 0.006). However, no significant differences were observed in sleep duration (OR: 0.61, 95% CI: 0.24–1.52, *p* = 0.295) or sleep patterns (OR: 0.73, 95% CI: 0.12–4.74, *p* = 0.727). Additionally, men who met physical activity guidelines reported lower risk of complaining of trouble sleeping (OR: 0.23, 95% CI: 0.09–0.56, *p* = 0.002) than men did not meet guidelines. However, no significant difference was observed between women (OR: 0.71, 95% CI: 0.27–1.88, *p* = 0.500). The results were presented in Table 3. In Figure 1, we used restricted cubic splines to flexibly model and visualize the relation of predicting the risk of complaining of trouble sleeping and time of moderate to vigorous PA per week in adults with ADHD who were receiving stimulant medicines. The plot showed a slight increase in the risk below 105 min/week (OR: 1.02, 95% CI: 0.92–1.14) that was not statistically significant and then started to decrease rapidly afterward (*P* for nonlinearity =0.025), with a substantial reduction in the risk approximately 341 min (OR: 0.87, 95% CI: 0.76–0.99) until approximately 1,250 min/week (OR: 0.60, 95% CI: 0.46–0.79), beyond which the curve leveled.

**Table 3:** Associations between physical activity level and sleep adjusting for covariates in adult ADHD patients with stimulant use.

	OR (95% CI)		*p* value
	Inactive group	Active group	
*Overall*			
Sleep duration	Reference	0.61 (0.24, 1.52)	0.295
Trouble sleeping	Reference	0.26 (0.10, 0.67)	0.006
Sleep patterns	Reference	0.73 (0.12, 4.74)	0.727
*Males*			
Sleep duration	Reference	0.47 (0.19, 1.13)	0.100
Trouble sleeping	Reference	0.23 (0.09, 0.56)	0.002
Sleep patterns	Reference	0.55 (0.09, 3.21)	0.500
*Females*			
Sleep duration	Reference	2.15 (0.83, 5.70)	0.120
Trouble sleeping	Reference	0.71 (0.27, 1.88)	0.500
Sleep patterns	Reference	1.70 (0.35, 12.5)	0.500

## Discussion

The present study presented a national sample of adults with ADHD who were using stimulant medicines to evaluate the relationship between physical activity and sleep. The motivation for this study was our previous work on this topic among children, but to date, this specific question has not been evaluated extensively in the adult population. Adults with ADHD using stimulant medicines are an especially vulnerable group that are frequently ignored and suffer from both ADHD and stimulants. It was not well-characterized whether physical activity would still be effective in this situation. The results of this study showed that adult ADHD patients on stimulant medications who met the general recommendation of physical activity guidelines (150 min/week of moderate to vigorous physical activity) in the US had a significantly lower risk of complaining of trouble sleeping. Additionally, a nonlinear dose–response relationship was found between physical activity and trouble sleeping, and a significant reduction in the risk of trouble sleeping was seen within the lower range until 341 min.

Adults frequently experience sleep issues, and the prevalence of insomnia disorder in adults is approximately 10–20%, with approximately 50% having a chronic course (27). The relationship between sleep disorders and ADHD is complex, with sleep disorders being both co-occurring conditions and consequences of ADHD. Despite the use of medication and comorbidities, there is abundant evidence that adults with ADHD are more prone to sleep problems, indicating that sleep deficits are intrinsic to ADHD. Adult ADHD is associated with longer objectively measured sleep onset latency, disrupted sleep maintenance, and later waking times. Adult ADHD patients with sleep onset problems may complain of daytime tiredness, which is related to a larger incidence of inattention symptoms (14, 28). Although the nature of the connection between ADHD and sleep problems is not fully known, inattention and impulsivity can be caused by sleep disturbances in the general population, suggesting that disturbed sleep may exacerbate ADHD symptoms (29). Conversely, extending sleep benefits to patients with ADHD in terms of attentional and behavioral functioning, emphasizing the importance of sleep as a target for intervention in this population (30), which should be given due weight. Clinically, psychostimulant drugs are commonly used for treating the core symptoms of ADHD by boosting dopamine and norepinephrine neurotransmitters in the brain. Numerous randomized controlled trials and meta-analyses have thoroughly demonstrated the effectiveness of these drugs (31–33). The therapeutic effects of stimulants are most likely related to the enhancement of central nervous system activity, particularly in brain regions that are critical for higher-order cognitive activities, such as the prefrontal cortex. However, many studies point out that stimulants do not improve and may even aggravate sleep problems in adults with ADHD (13, 14). This could be ascribed to the side effects of stimulants, which depend on the specific drug used, dosages, and individual characteristics of the patient. It is critical to realize that stimulant drugs should be used with other management strategies, such as habit modification or anxiety reduction, to counteract the negative effects on sleep.

According to the most recent research, which was published in 2023, adhering to the 24-h movement behavior guidelines, which call for getting enough sleep, doing at least 60 min of moderate-to-vigorous physical activity each day, and limiting recreational screen time to no more than 2 h per day was linked to a lower risk of cognitive and social difficulties in children and adolescents with ADHD (34). This study highlights the importance of physical activity, which appears to have a positive impact on social and neurocognitive function in children and adults. Clinical trials of children with ADHD and preclinical studies on spontaneously hypertensive rats, an animal model of ADHD, have shown that aerobic exercise can be helpful as an adjunct treatment to medication for ADHD. Some studies have also indicated that physical activity can be a helpful tool in treating sleep-related problems in children with ADHD (35), and aerobic exercise may act on catecholamine pathways as a stimulant medication (36). Physical activity can effectively shorten the time it takes to fall asleep, increase total sleep time, and improve sleep quality for many people. Nevertheless, no controlled clinical trials in adults with ADHD have been conducted on the efficacy of physical activity for improving sleep in adults with ADHD; we performed a cross-sectional analysis to demonstrate the efficacy of PA for improving sleep among stimulant users of ADHD. Our results suggest that greater amounts of weekly moderate-to-vigorous activity result in a lower risk of trouble sleeping, indicating that maintaining a certain amount of PA is a protective factor to sleep for adult ADHD with stimulant medication use. This highlights the importance of physical activity in adhering to healthy lifestyle behaviors. Activity duration is a significant parameter in exercise intervention trials. In this study, it was proven that the accumulation of moderate-to-vigorous physical activity within a week for a certain period of time had a positive effect on sleep, as recommended by the guidelines, but we found that a large number of people spent much more than 150 min among the included adult ADHD participants, and the significant effect could be attributed to this.

It was worth mentioning that there was a significant difference in men, but was not significant in women. Possible explanations for this discrepancy were likely physiological differences and hormonal levels (37, 38). There are physiological differences between men and women, including differences in sleep structure and regulatory mechanisms. These differences may contribute to a greater positive response to physical activity in men, while women may exhibit a lesser response to physical activity. In addition, women experience hormonal fluctuations during different physiological cycles, such as the menstrual cycle and menopause. These hormonal changes may influence women’ response to physical activity and the effectiveness of improving their sleep quality. In a detailed dose–response plot, we found that it takes at least 341 min of moderate-to-vigorous physical activity per week to have a significant positive impact on sleep. The results may be used by researchers and clinicians to develop intervention models for individuals with ADHD that focus on the mediating functions of lifestyle factors to enhance sleep quality (39). Moreover, a dose–response plot portrayed an initial surge in the risk of trouble sleeping (0–105 min of physical activity), and it would be beneficial for elaborating on it. When individuals engage in limited or irregular physical activity, their bodies may require adaptation to the new physical demands (40). This adaptive process involves physiological adjustments and repairs, which can momentarily disrupt sleep quality (40). However, this phenomenon is usually temporary, and sleep quality typically improves once the body successfully adapts and adjusts to the new physical activity load. As such, it is crucial to establish a consistent physical activity regimen and allocate adequate time for the body to acclimate to the novel physical demands. However, these findings require confirmation through longitudinal and interventional studies with large sample sizes because there are few controlled clinical trials on sleep problems in people with ADHD. Additionally, more clinical study is also needed on the clinical impact of exercise on sleep problems in adults with ADHD to explore potential shared neurobiological mechanisms.

Our study is significant because it provides clear evidence of an association between sleep and physical activity in adults with ADHD on stimulant medication population. Nevertheless, we must acknowledge several shortcomings and limitations in our study. Firstly, although this article represented one of the first attempts to investigate the role of physical activity with the associations of sleep among adults with ADHD on stimulant medications population, we cannot rule out that some stimulant medications may have positive effects on sleep as various forms of stimulants were used in the current study. Moreover, it was a cross-sectional study and we cannot prove causality or determine whether our findings were due to direct effects of physical activity on sleep. In order to expand on the findings, future studies need to consider these. Secondly, research on sleep in adults with ADHD has recently focused on the role of circadian rhythm and circadian preference (chronotype), and this study did not provide that relevant information, and these outcomes deserve further exploration through sleep monitoring devices in clinical trials. Thirdly, data on sleep and physical activity were subjectively self-reported, which could be improved in future studies by using objective measures such as polysomnography and actigraphy.

## Conclusion

Our findings from the NHANES revealed associations and a dose-effect relationship between physical activity and sleep condition in the adult ADHD patients who were using stimulant medications, and strategies to optimize sleep should be recommended for adults with ADHD using stimulants, including increasing physical activity as a healthy lifestyle choice. Gender difference should be considered as an important factor for further studies to examine these associations and explore potential mechanisms. It is important to confirm our findings through additional longitudinal and interventional studies to explore the effect of various exercises on sleep quality in populations with adult ADHD who are taking stimulants.

## Data availability statement

The original contributions presented in the study are included in the article/supplementary material, further inquiries can be directed to the corresponding author.

## Ethics statement

The study analyzed data downloaded from the National Health and Nutrition Examination Survey public database. The National Center for Health Statistics of the Centers for Disease Control and Prevention Institutional Review Board approved the protocol for the original NHANES data collection, and informed consent was acquired from all individual participants (https://www.cdc.gov/nchs/nhanes/irba98.htm).

## Author contributions

FZ and YR conceived and designed the study. FZ and DK collected the data and performed the analysis. XZ, BL, YS, TQ, and YY assisted with the investigation. FZ and BL wrote and revised the manuscript. All the authors edited and approved the manuscript.

## Footnotes

^1^ https://www.cdc.gov/nchs/nhanes/index.htm

^2^ https://www.cdc.gov/nchs/nhanes/irba98.htm
